# Production and Purification of High-Titer Newcastle Disease Virus for Use in Preclinical Mouse Models of Cancer

**DOI:** 10.1016/j.omtm.2017.10.004

**Published:** 2017-10-16

**Authors:** Lisa A. Santry, Thomas M. McAusland, Leonardo Susta, Geoffrey A. Wood, Pierre P. Major, Jim J. Petrik, Byram W. Bridle, Sarah K. Wootton

**Affiliations:** 1 Department of Pathobiology, University of Guelph, Guelph, ON N1G 2W1, Canada; 2 Juravinski Cancer Centre, 699 Concession Street, Hamilton, ON L8V 5C2, Canada; 3 Department of Biomedical Sciences, University of Guelph, Guelph, ON N1G 2W1, Canada

**Keywords:** Newcastle disease virus, oncolytic virus, tangential flow filtration, preclinical grade, intravenous, allantoic fluid

## Abstract

Newcastle disease virus (NDV) is a single-stranded, negative-sense RNA virus in the *Paramyxoviridae* family. Although primarily an avian pathogen, NDV is a potent oncolytic virus that has been shown to be safe and effective in a variety of preclinical cancer models and human clinical trials. To produce virus for oncolytic trials, NDV is commonly amplified in embryonated chicken eggs and purified from the allantoic fluid. Conventional methods for purifying virus from allantoic fluid often result in relatively low-titer preparations containing high levels of impurities, including immunogenic chicken host cell proteins from allantoic fluid. However, large quantities of virus need to be delivered intravenously to administer oncolytic NDV systemically to mice. This route of administration requires virus preparations that are both highly concentrated (to enable delivery of small volumes) and highly pure (to limit toxic effects from contaminants). Given the accumulation of promising preclinical and clinical data demonstrating the efficacy of NDV as an oncolytic agent, strategies for increasing the titer and purity of NDV preparations are sorely needed to allow for effective intravenous administration in mice. Here, we describe an optimized protocol for the rescue, production, and purification of high-titer *in vivo*-grade NDV for preclinical studies in mouse models.

## Introduction

Newcastle disease virus (NDV) is the causative agent of Newcastle disease, an economically important disease of poultry. NDV belongs to the order Mononegavirales, family *Paramyxoviridae*, and genus *Avulavirus*. In mammals, NDV selectively replicates in and lyses cancer cells, while sparing normal cells, because of defects in anti-viral and apoptotic pathways common to many cancers.^1^, ^2^ In addition to direct oncolysis, NDV possesses strong immunostimulatory properties that can overcome cancer-induced immunosuppression and generate effective anti-tumor immune responses.^3^, ^4^, ^5^ In the years since its oncolytic potential was discovered,^6^ NDV has been evaluated in multiple clinical trials with promising results^7^, ^8^, ^9^, ^10^, ^11^ and a remarkable safety profile.^12^ Further, with the advent of reverse genetics, NDV recombinants with enhanced oncolytic and immunostimulatory properties have been engineered.

Given the importance of these clinical applications of NDV, there has been considerable interest in developing optimized methods for engineering, producing, and purifying NDV for use *in vivo*. NDV is typically rescued in human cell lines, propagated in embryonated chicken eggs, and harvested from the allantoic fluid. Propagation in cell culture is possible; however, virus yields are lower.^13^, ^14^ Regardless of the method used to produce NDV, purification and concentration of the virus is required to remove host contaminants from the allantoic fluid or cell culture media. Furthermore, high-titer ultrapure virus is needed for systemic intravenous delivery, which requires purity beyond what is commonly needed for intra-tumoral inoculation or vaccination.

Methods used in the purification of clinical-grade viruses include anion exchange chromatography, affinity chromatography, density-gradient ultracentrifugation, and tangential flow filtration (TFF).^15^, ^16^ Typically, these methods are tailored to specific viruses and require careful optimization. Indeed, a recent review by Ungerechts et al.^15^ highlights the difficulty and challenges associated with purification of clinical-grade viruses, including variation in particle size and morphology, which for NDV ranges from 100 to 500 nm in diameter with spherical (most common) and filamentous forms.^17^, ^18^ Given the complexities of producing ultra-pure virus, and the paucity of information in the literature describing methods for purifying NDV, we have optimized a laboratory-scale protocol for production of ultraclean, high-titer, preclinical-grade NDV that can be safely administered intravenously to tumor-bearing mice at unprecedented titers of 1 × 10^9^ plaque-forming units (PFUs) per dose.

## Materials

### Reagents

• GenElute HP Plasmid Maxiprep Kit (NA0300S; Sigma-Aldrich)
• PolyJet (SL100688; SignaGen)
• High Glucose DMEM
• PBS without calcium/magnesium/phenol red
• Trypsin
• Bovine calf serum (BCS)
• 200 mM L-glutamine
• Sucrose
• Polyethylene glycol, average molecular weight number (M_n_) 20,000 (PEG-20) (81300; Sigma-Aldrich)
• Chicken red blood cells (RBCs) in Alsever’s solution (1:1)
• Specific pathogen-free (SPF) embryonated chicken eggs
• BALB/c mice
• Ethanol
• Standard PCR reagents
• Iodine
• Nail polish

### Equipment

• 10-cm, 35-mm, 6-well, 96-well tissue culture plates
• 96-well V-bottom plates
• Digital 1502 Sportsman egg incubator (1,502 W; humidity kit, 3030; Berry Hill)
• Egg candler (A46; Berry Hill)
• 1- and 3-mL syringes
• 25G ± 5/8 in and 18G needles, 21G blunt needles
• Fine-point forceps
• Surgical scissors
• Masterflex L/S Digital Drive and Masterflex L/S Easy-Load II Head for Precision Tubing (07522-20 and -60; Cole Parmer)
• Supracap 50 Depth Filter Pall V100P (SC050V100; Pall Laboratory)
• Omega Membrane LV Centramate Cassette, 100K (OS100T02; Pall Laboratory)
• Centramate Cassette Holder (CM018V; Pall Laboratory)
• Tubing screw clamp (88216; Pall Laboratory)
• Utility pressure gauge (×3) (68355-06; Cole-Palmer)
• Male and female Luer-Lok × 1/8 in national pipe thread (NPT; 41507-44 and 46; Cole-Palmer)
• Female threaded tee fittings, nylon, 1/8 in NPT(F) (06349-50; Cole-Palmer)
• Masterflex C-Flex ULTRA tubing, L/S 16, 25 ft (06434-16; Cole-Palmer)
• 10K molecular weight cut off (MWCO) Slide-A-Lyzer Dialysis Cassettes (12 mL) (66451; Thermo Fisher)
• Thinwall Ultra-Clear ultracentrifuge tubes (344059; Beckman Coulter)
• Centrifuges (ultracentrifuge, high speed, clinical)
• Fluorescent microscope
• Biological safety cabinet
• CO_2_ incubator
• Multichannel pipette
• Thermocycler

### Reagent Setup

#### Plasmids

The full-length cDNA genome plasmid of LaSota NDV-GFP and helper plasmids (pTM1-NP, pTM1-P, and pTM1-L) were a kind gift from Dr. Peter Palese (Mount Sinai, NY, USA). Helper plasmids and the NDV-GFP genome plasmid were purified using the GenElute HP Plasmid Maxiprep Kit.

#### Cells and Viruses

HEp-2 (CCL-23), DF-1 (CRL-12203), and BHK-21 (CCL-10) cells were maintained in DMEM supplemented with 10% BCS and 2 mM L-glutamine (complete DMEM [cDMEM]) at 5% CO_2_ and 37°C. Modified vaccinia Ankara virus expressing T7 RNA polymerase (MVA-T7)^19^ was a kind gift from Dr. Bernard Moss. MVA-T7 was propagated and titrated in BHK-21 cells.^20^

#### Embryonated Chicken Eggs

SPF embryonated chicken eggs were maintained at 37°C and 60% humidity, and turned once every hour from days 0 to 9. Following inoculation with NDV, eggs were no longer turned.

#### Chicken RBCs

Chicken RBCs in Alsever’s solution (1:1) were stored at 4°C. RBCs were prepared for hemagglutination assay (HA) by removing 2 mL of whole blood, washing with 13 mL of PBS, and pelleting at 500 × *g* for 10 min. Supernatant was removed with a pipette and discarded, and the wash step was repeated twice. Pelleted RBCs were diluted in 1% BCS-PBS to generate a 0.5% working stock.

### Equipment Setup

**Figure 1:**
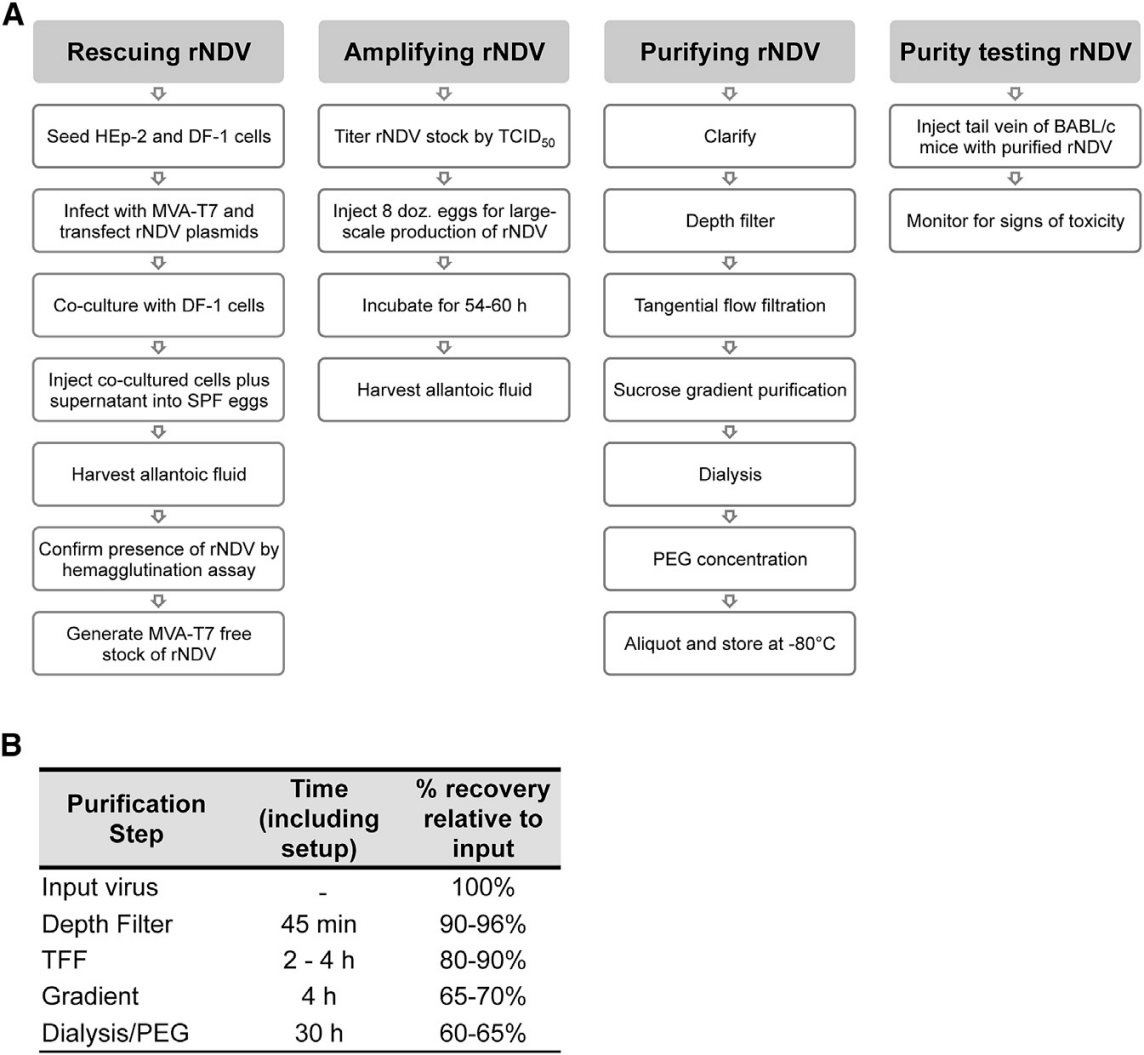
Overview of NDV Purification Procedure and Percent Recovery at Individual Steps (A) Chart outlining the various steps involved in generating high-titer preclinical-grade rNDV. (B) Percent recovery at each step in the NDV purification process.

A detailed description of how to set up the equipment for each step in the purification procedure (see Figure 1) is described in the following section.

## Procedure

For an overview of the NDV purification procedure, see Figure 1. Note that although there are published protocols available for rescuing recombinant NDV (rNDV),^21^, ^22^ one of the aims of this paper was to provide a comprehensive protocol for both the production and the purification of high-titer NDV. Because oncolytic NDV is designated as a Risk Group 2 pathogen in Canada, the procedures described in this methods paper were conducted in a BSL-2 laboratory within a type IIA biological safety cabinet. Importantly, all production and purification steps must be conducted in a type IIA biological safety cabinet for sterility and safety purposes.

### 1 Rescuing rNDV from Plasmid DNA

#### 1.1 Initiate Incubation of Fertile Chicken Eggs (Day 0)

Because 0-day-old embryonated chicken eggs will be inoculated, begin incubating eggs 3 days before initiating the 5-day cell culture component of the virus rescue.

#### 1.2 Seeding of HEp-2 Cells (Day 4)

Seed 1 × 10^6^ HEp-2 cells/well in a six-well plate (two wells for controls and four wells for rescue) and incubate overnight to let cells adhere. Cells should be approximately 80%–90% confluent the following day.

Seed 3 × 10^6^ DF-1 cells into a 10-cm dish such that they will be 90% confluent and ready for co-culturing in 2 days.

#### 1.3 Infection/Transfection (Day 5)

Transfect cells at the beginning of the day so that medium can be changed within 5–8 hr. Wash HEp-2 cells with PBS and infect each well with MVA-T7^19^ at an MOI of 1 in a final volume of 300 μL (diluted in basal DMEM [bDMEM]). To optimize adsorption, place the six-well plate on a rocker or manually redistribute inoculum every 10 min for 1 hr. After adsorption, aspirate inoculum, wash cells twice with PBS, add 1.5 mL of cDMEM, and return the plate to the incubator.

**Figure 2:**
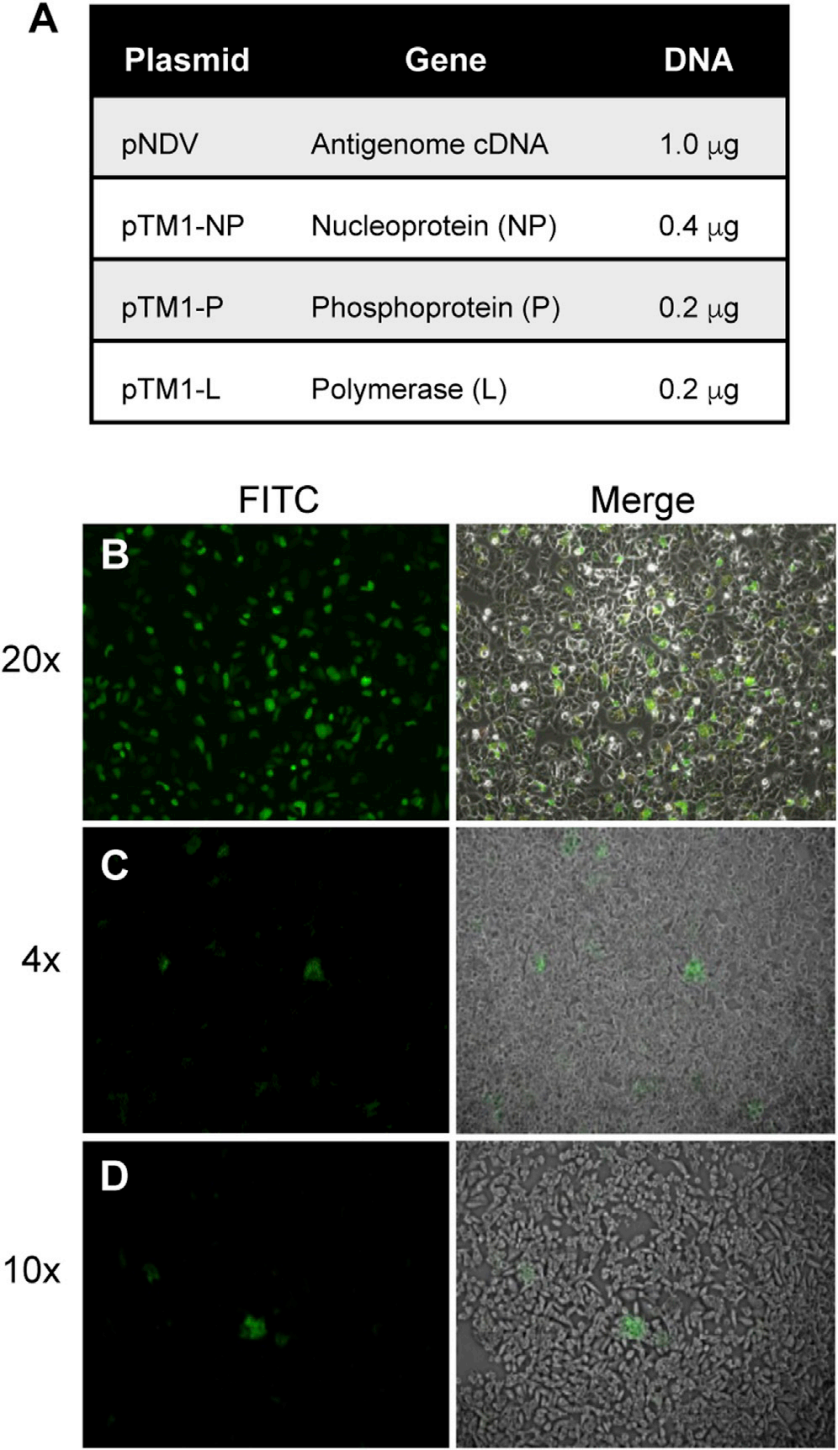
Specifications for rNDV Rescue and Co-culture (A) Table listing the four plasmids transfected into HEp-2 cells to produce rNDV. The NDV antigenome and helper genes are under the control of a T7 promoter. The amount of plasmid DNA required to transfect one well of a six-well tissue culture dish using the PolyJet transfection reagent is shown. (B) An image of HEp-2 cells 48 hr after transfection with 1 μg of pSIN-GFP using the PolyJet transfection reagent. (C) 4× and (D) 10× representative fluorescent (left) and merged bright-field (right) images of HEp-2 cells transfected with plasmids for rescuing rNDV expressing GFP and then co-cultured with DF-1 cells for 72 hr.

For transfection of HEp-2 cells, PolyJet was found to be the most efficient and cost-effective transfection reagent (Figure 2B). Set up the control for transfection efficiency by adding 1 μg of pSin-EGFP (or any mammalian expression vector encoding a fluorescent reporter gene) to 50 μL of bDMEM. Next, to rescue rNDV, add 0.4 μg of pTM1-NP, 0.2 μg of pTM1-P, 0.2 μg of pTM1-L, and 1 μg of genome plasmid to 50 μL of bDMEM (Figure 2A). For each well of cells to be transfected, dilute 3 μL of PolyJet into a separate tube of 50 μL of bDMEM. Add 50 μL of PolyJet dilution to a 50 μL DNA solution (rescue and control) *(do not reverse this order*), pipette up and down immediately, and incubate at room temperature (RT) for 10–15 min. Add 100 μL/well transfection mixture drop-wise to the MVA-T7-infected HEp-2 cells; rock the plate for proper distribution. Incubate cells for 5–8 hr, remove the transfection mixture, and replace with 1.5 mL of cDMEM.

#### 1.4 Co-culturing with DF-1 Cells (Day 6)

Because NDV replicates well in continuous chicken embryo fibroblast DF-1 cells, a co-culture step is included to amplify rNDV. 24 hr post-transfection/infection, wash wells containing HEp-2 cells (excluding the transfection control) with PBS, detach cells with 0.5 mL of trypsin, neutralize with 1 mL of cDMEM, and add the mixture from one well to one 10 cm dish. Immediately wash a confluent 10 cm dish of DF-1 cells with PBS, detach using 1.5 mL of trypsin, neutralize with 8.5 mL of cDMEM, then pellet cells at 500 × *g* for 5 min. Suspend the DF-1 cell pellet in 15 mL of cDMEM and add 3.5 mL to each of four 10 cm dishes containing the HEp-2 cells from the infection/transfection rescue (1.5 mL HEp-2 cells + 3.5 mL DF-1 [∼1:1 ratio] for a final volume of 5 mL) and incubate for 72 hr. Cells are co-cultured in a reduced volume to concentrate virus. After 24–48 hr, check for EGFP expression in the transfection control well to confirm that the transfection was successful.

#### 1.5 Injecting Co-cultured Cells into SPF Eggs (Day 9)

After 72 hr of co-culturing, collect and save supernatant (contains majority of virus). Wash cells with PBS and detach adherent cells with 750 μL of trypsin. Add the original supernatant back to cells to neutralize the trypsin (it is not necessary to remove trypsin). If eggs are not ready for inoculation, the cell-supernatant mixture containing rNDV can be stored at 4°C overnight (O/N) or −80°C for longer-term storage; however, the efficiency of virus rescue will decrease with each freeze-thaw.

**Figure 3:**
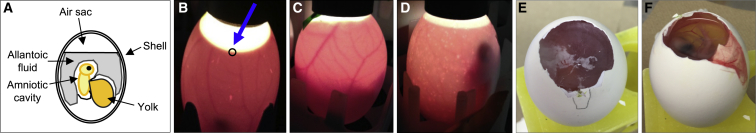
NDV Infection and Virus Harvest from Embryonated Eggs (A) Diagram of an embryonated chicken egg on day 9, showing the location of the air sac, allantoic fluid, yolk sac, and embryo. (B) Image showing the location of the air sac in a day 9 embryo when candled. The small black circle indicates where a hole should be made for the injection, at the interface between the air sac and the allantoic fluid, in a region devoid of large vasculature. (C) Example of a healthy day 11 embryo imaged 48 hr post-inoculation with low-titer NDV. Presence of healthy vasculature and movement of the embryo are signs that the embryonated egg is alive. (D) Example of a dead day 11 embryo imaged 48 hr post-inoculation with high-titer NDV. There is an absence of vasculature and no movement from the embryo. The embryo appears dark as a result of the lack of movement. (E) Embryonated egg in which the apical side of the egg has been removed using forceps and surgical scissors to reveal the chorioallantoic membrane. (F) Chorioallantoic membrane cut and peeled back to reveal the allantoic cavity from which the allantoic fluid is collected.

Candle 9-day-old embryonated eggs to locate the interface between the air sac and chorioallantoic cavity (Figure 3A). Approximately 2 mm above this interface, identify an injection site that will not cause perforation of the vasculature, and mark with a pencil (Figure 3B). Clean eggs at the site of injection using 10% iodine in 70% ethanol and carefully pierce the shell with sharp tweezers. Inject 250–300 μL of the supernatant+cell mixture into the chorioallantoic cavity using a 1 mL syringe and 25G needle. Inject six eggs/well of rNDV that you are attempting to rescue. Six eggs are inoculated to account for low virus titer and loss of eggs because of mechanical error. After injection, cover puncture site with nail polish and return egg to incubator. Monitor eggs for death at 24 hr and then twice a day thereafter. Look for loss of vasculature in the chorioallantoic membrane and lack of movement from the embryo (Figure 3D). Discard embryos that die <24 hr post-inoculation. Eggs that die after 24 hr are moved to 4°C to minimize autolysis. At 96 hr post-inoculation, the remaining eggs should be moved to 4°C. Harvest allantoic fluid within 12 hr of egg chilling.

#### 1.6 Harvesting Allantoic Fluid (Days 11–13)

Clean the top of the egg with 70% ethanol. Starting at the site of injection, use sterile forceps and surgical scissors to break open the apical side of the egg where the air sac is located, revealing the chorioallantoic membrane (Figure 3E). Peel back the membrane, exposing the allantoic cavity (Figure 3F). Using forceps, push the embryo down and carefully collect the allantoic fluid with a 10 mL serological pipette and place into a 15 mL conical tube. Clarify the allantoic fluid by centrifugation at 1,500 × *g* for 10 min at 4°C. Transfer supernatant into a new tube using serological pipet. Do not pool the allantoic fluid from individual eggs because each sample will be tested for the presence of virus separately.

#### 1.7 HA (Days 11–13)

**Figure 4:**
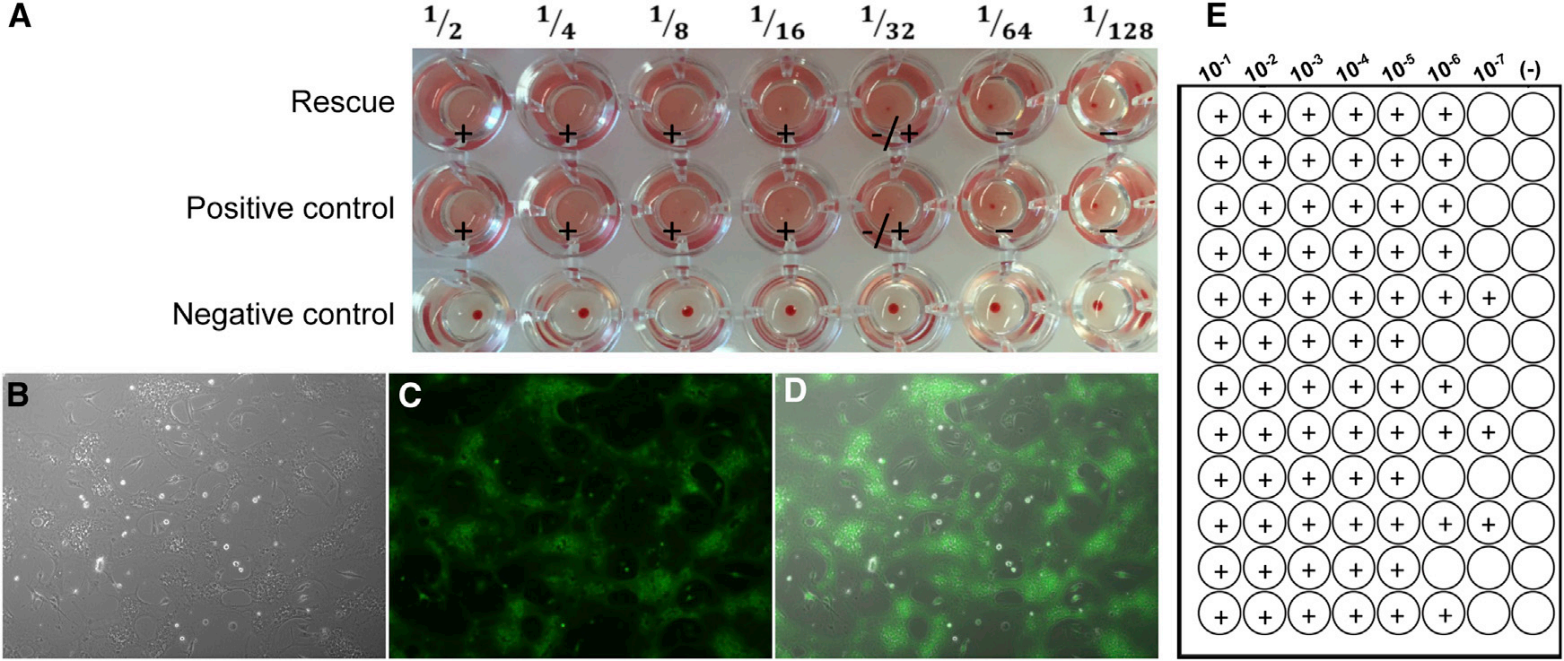
Typical Hemagglutination Assay Results Showing Successful Rescue of rNDV Expressing GFP (A) Example of a hemagglutination assay (HA). In the negative control sample (lacking virus), RBCs form a distinct pellet at the bottom of the well. In the positive control (allantoic fluid containing NDV), RBCs form a diffuse lattice-like structure. For the sample shown, RBCs begin to button at the 1/32 dilution and appear negative in the 1/64 dilution, representing an HA titer of 32. (B–D) Bright (B), fluorescent (C), and merged (D) images of DF-1 cells infected with 10 μL of allantoic fluid containing rNDV-GFP at 24 hr post-infection. Note that in DF-1 cells, rNDV causes the formation of large multinucleated cells or syncytia. (E) Example of TCID_50_ plate setup and scoring. In the 10^−1^ to 10^−5^ lanes all 12 replicates are positive, 10^−6^ has 9/12 positive wells, and 10^−7^ has 3/12 positive wells. Using the Spearman-Karber calculation for TCID_50_, the virus titer is 3.16 × 10^8^ TCID_50_/mL or 2.18 × 10^8^ PFUs/mL.

Perform HA assay to confirm the presence of rNDV in allantoic fluid (Figure 4A). An allantoic fluid negative control and an NDV or influenza virus positive control should be included in the assay. Pipet 100 μL of PBS into each well of a 96-well V-bottom plate. Next, pipet 100 μL of allantoic fluid from individual eggs into a well in the first column of the plate. Perform 2-fold serial dilutions across the plate. Suspend RBCs and pipette 100 μL of 0.5% RBCs into each well. Incubate plate at RT for 30–45 min or until a “button” forms in the negative control well. Titer is expressed as HA units and is determined as the inverse of the last dilution of virus showing complete agglutination (e.g., agglutination at a dilution of 1/32 = 32 HA units). Store allantoic fluid at 4°C for up to 2 days, otherwise freeze at −80°C. If the HA test is negative, repeat rescue.

#### 1.8 Removing MVA-T7 from Virus Stock

Residual MVA-T7 should be eliminated before production of a working stock of rNDV. Select allantoic fluid from three positive eggs and 0.45 μM filter to remove MVA-T7. Set up a 10-fold dilution series for each positive sample, diluting up to 10^−6^ in PBS. Inject 100 μL of filtered, diluted rNDV into day 9 SPF eggs. Inject three eggs per dilution from 10^−3^ to 10^−6^ for a total of 12 eggs. Monitor eggs for 96 hr (discard eggs that die in <24 hr). Expect death between 48 and 96 hr, and move dead eggs to 4°C. Confirm the presence of rNDV in allantoic fluid by HA assay. Select rNDV-containing allantoic fluid from eggs that were inoculated with the highest dilution and use this stock for large-scale amplification of virus. Note that this step is a limiting dilution for MVA-T7, which has mostly been removed by filtering but can allow for selection of a single clone of rNDV. Confirm removal of MVA-T7 by infecting a 35 mm plate of confluent BHK-21 cells with 10 μL of allantoic fluid from your stock virus. Isolate genomic DNA after 48 hr and perform PCR using primers for the B18R gene of MVA-T7 (forward [Fwd]: 5′-ATGACGATGAAAATGATGGTACATA-3′ and reverse [Rv]: 5′-CTCCAATACTACTGTAGTTGTAAGG-3′). If MVA-T7 is present, repeat filtration and limiting dilution steps.

### 2 Amplifying rNDV

#### 2.1 Titering rNDV by 50% Tissue Culture Infective Dose

Once an MVA-T7-free stock of rNDV has been selected, determine the 50% tissue culture infective dose (TCID_50_). Based on Poisson distribution, TCID_50_ values can be converted to PFUs according to the formula 1 TCID_50_ = 0.69 PFU.^23^ Seed 96-well plates with 4 × 10^4^ DF-1 cells/well in 100 μL of cDMEM. The next day, prepare seven 1.5 mL microcentrifuge tubes containing 900 μL of PBS and perform 10-fold serial dilutions of stock rNDV containing allantoic fluid up to 10^−7^. Transfer 10 μL of each dilution to a 96-well plate containing DF-1 cells in 12 replicates (Figure 4E). Five days post-infection, score the plate by marking a plus sign (“+”) on each well where cytopathic effects are visible. Determine titer using the Spearman-Karber calculation.^24^

#### 2.2 Injecting Eggs for Large-Scale Production of rNDV

**Figure 5:**
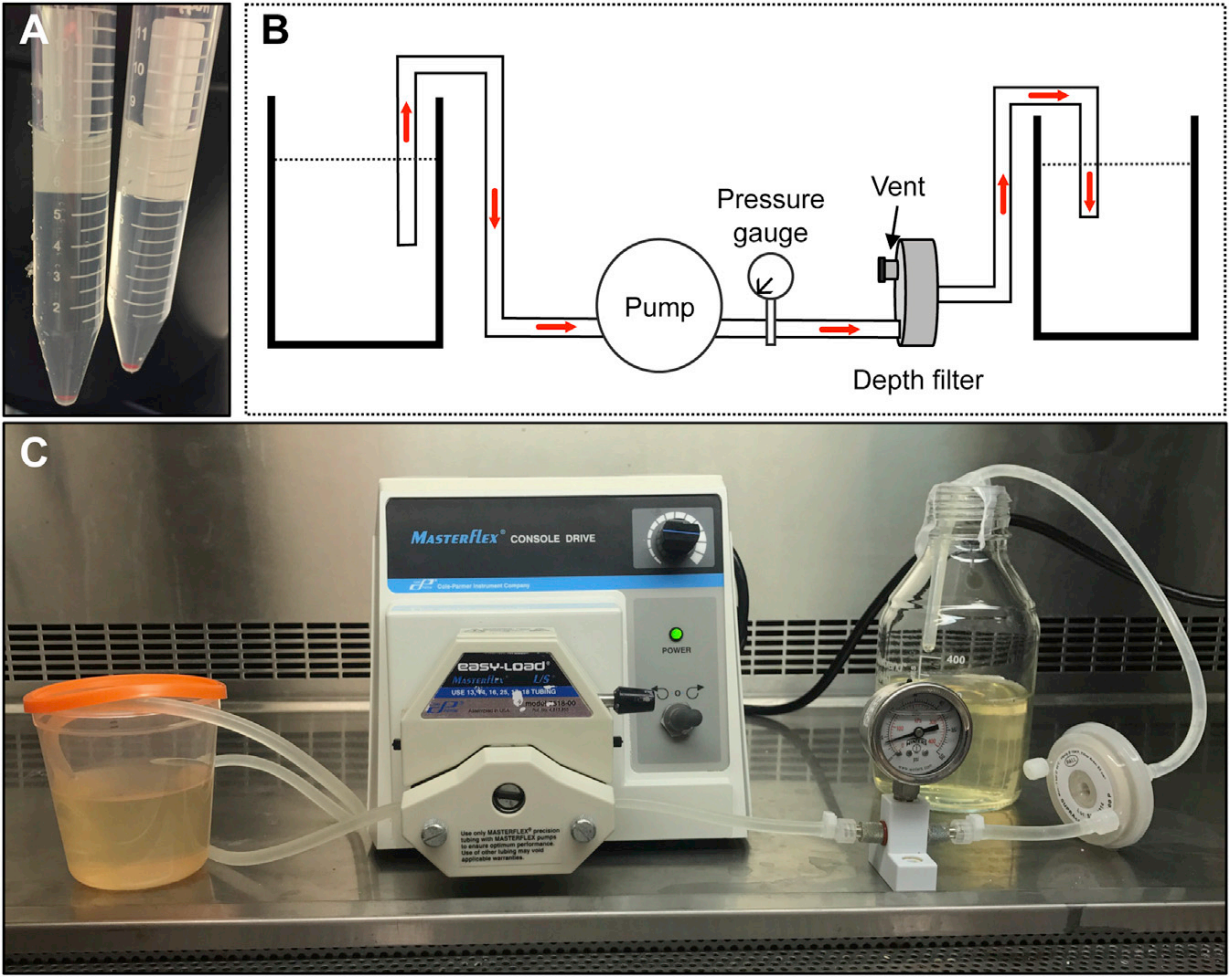
Depth Filtration Setup (A) An example of contaminant-free allantoic fluid suitable for TFF. (B) Schematic of a typical depth filtration setup. (C) Image of a batch of NDV containing allantoic fluid being depth filtered. Note the placement of the pressure gauge. Allantoic fluid is filtered into a sterile container.

For production of large preclinical batches of rNDV, inoculate a minimum of eight dozen 9-day-old embryonated chicken eggs with 500 PFUs in 100 μL/egg. At 45–50 hr post-inoculation (or 72 hr in the case of lentogenic strains), regardless of viability, move all eggs (excluding any that died at <24 hr) to 4°C for 2 hr. It is imperative that the allantoic fluid be as clean as possible and free of contaminating albumen and yolk (Figure 5A). Discard allantoic fluid that is contaminated with yolk or other viscous liquids.

### 3 Virus Purification

Importantly, because tubing is stored in 1 M NaOH, run sterile water or PBS through the tubing to avoid inactivating virus.

#### 3.1 Depth Filtration

Clarify NDV-containing allantoic fluid by centrifugation at 1,500 × *g* for 10 min at 4°C. If necessary, virus can be frozen after clarification, but not before. Pool clarified allantoic fluid and filter through a Supracap 50 Depth Filter (Pall Laboratory) (see Figures 5B and 5C for setup). Before attaching the depth filter, sterilize tubing by running 100 mL of 1 M NaOH followed by 300 mL of Milli-Q water through the tubing. Prime the lines by running 50 mL of PBS through and stopping when there is 5 mL left in the tube. Attach depth filter to the tubing, remove venting cap from the depth filter, and flow 50 mL of PBS through until the air is displaced and PBS begins to overflow out the top. Re-cap and continue to flow PBS through. Although minor air bubbles are acceptable, avoid introducing excessive air or the venting process will need to be repeated. Stop the pump when 5 mL of PBS remains and the lines and filter contain PBS. Next, pump virus through the depth filter, maintaining a pressure of <10 psi to avoid shearing the virus. If pressure increases, reduce the flow rate. Once virus-containing allantoic fluid has passed through the depth filter, maximize retrieval by running 50 mL of 5% sucrose-PBS through the depth filter and collect until dry. Virus is now ready for TFF. If necessary, virus can be stored at 4°C overnight. A minimal amount of virus (<6%) is lost in the depth filtration step (Figure 1B). To sanitize tubing before storing, run 100 mL of 1 M NaOH through and store in 1 M NaOH.

#### 3.2 TFF

TFF is a highly efficient method for concentrating large volumes of virus-containing supernatant and for buffer exchange purposes.

#### 3.2.1 Installing TFF Cassette

**Figure 6:**
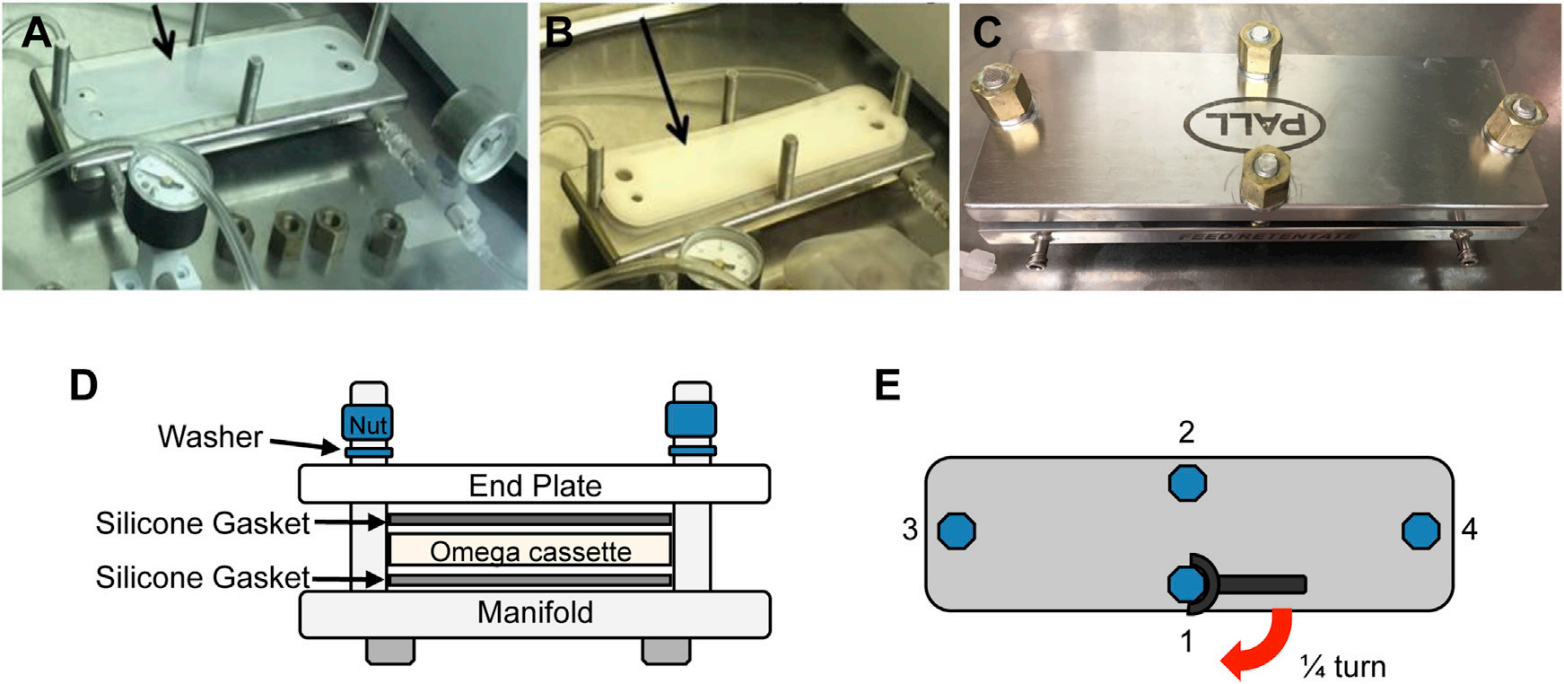
Assembly of TFF Cassette (A) Bottom plastic gasket placed on top of the manifold. (B) Cassette placed on top of bottom gasket, followed by another plastic gasket on top (not shown). (C) Final Centramate setup with end plate and nuts in place. (D) Side view of the final Centramate setup showing placement of the two gaskets and the cassette. (E) A representation of the order in which to tighten bolts 1 → 4. Tighten each nut by one-fourth of a turn each time so as to avoid uneven pressure on one section of the cassette and subsequent damage.

Remove retaining nuts and washers from the Centramate device (Figure 6) and then remove the end plate from the base of the Centramate manifold. Remove the Centramate cassette (stored in 0.3 M NaOH) from the packaging and inspect for damage or presence of foreign material that could affect sealing of the cassette. Rinse the cassette and plastic gaskets with sterile deionized water. Position a gasket flat against the bottom of the manifold (Figure 6A) and then place the cassette and a second gasket on top (Figure 6B), all the while ensuring that the holes in the manifold, gaskets, and cassette line up. Place the end plate on top of the cassette sandwich. Place washers on each bolt, then tighten nuts by hand, applying even pressure (Figures 6C and 6D). Using a torque wrench set to 6–7 Nm, tighten nut (#1) one-fourth of the way, then proceed to the opposite nut (#2) and tighten one-fourth of the way (Figure 6E); repeat for the remaining two nuts. This step is important because it ensures an even distribution of pressure across the cassette. Stop tightening each nut when the torque wrench “clicks,” indicating that the correct torque has been reached. Notably, tightening beyond the suggested torque can damage the cassette.

#### 3.2.2 Sanitizing the Cassette and Tubing

**Figure 7:**
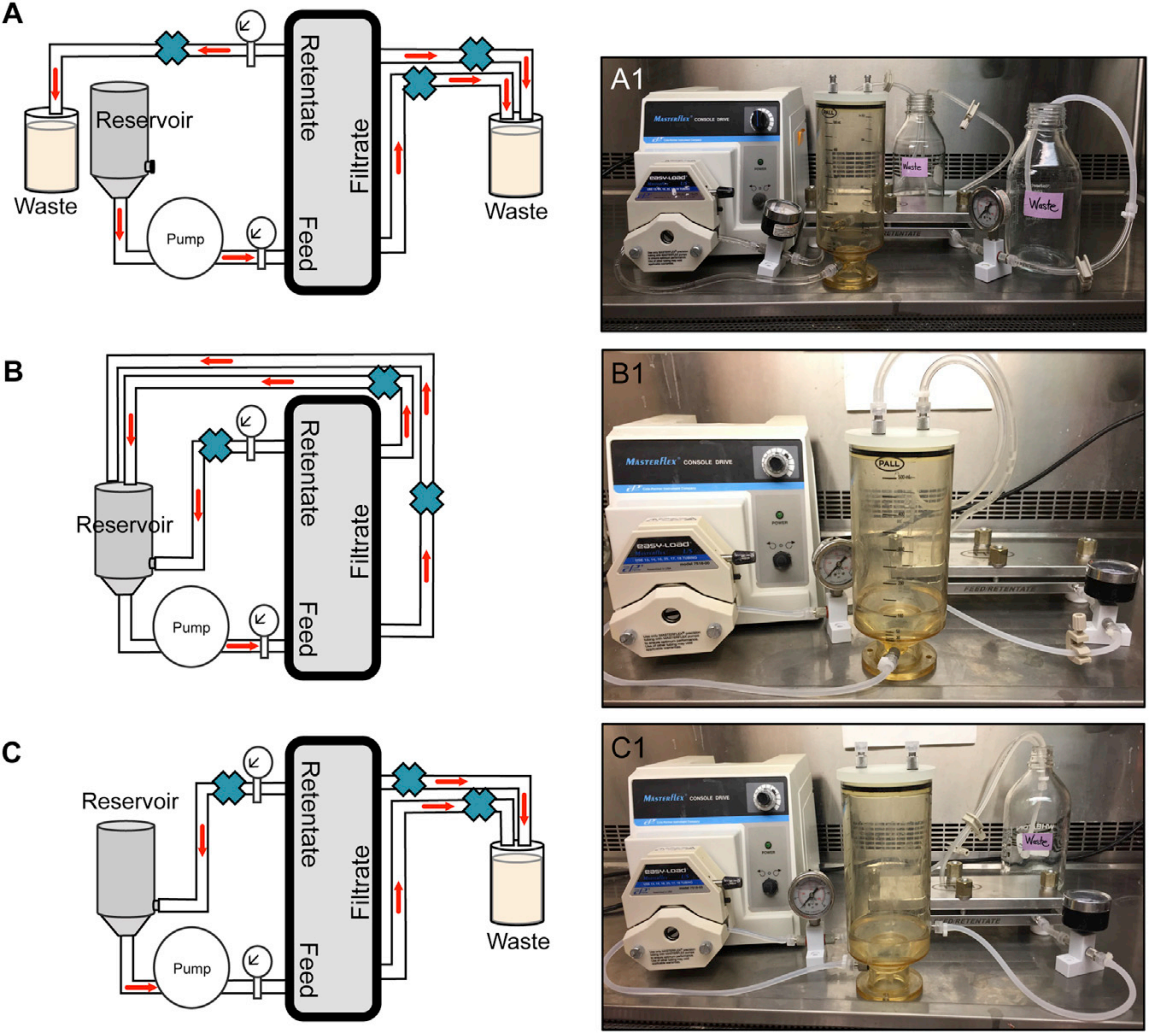
Schematic of the Three Different TFF Configurations Utilized for NDV Purification (A) Schematic of the TFF setup for flushing and cleaning the cassette and tubing. Note placement of pressure gauges. Labels on the side of the Centramate indicate where the feed/retentate and filtrate lines should be connected. (B) Setup for a closed circulating system. The filtrate and retentate lines go back to the reservoir. Blue crosses indicate where clamps can be attached. These clamps can be used to increase pressure during sanitation cycles. (C) Schematic of virus purification and concentration setup. Note the retentate containing the virus is circulating back into the reservoir, whereas the filtrate line ends up in a waste container.

Using clear LS-16 Masterflex tubing and connectors that have been flushed with sterile water to remove storage solution, attach the Centramate, reservoir, and two pressure gauges as shown in Figure 7A. Once connected, inspect the system for leaks, particularly at tubing connections. Zip ties can be used to secure tubing connections. To sanitize the system, fill the feed reservoir with 300 mL of 0.3 M NaOH. Direct the retentate and filtrate lines to drain into a waste container (Figure 7A). Adjust the pump to a flow rate of 10–20 psi, but do not exceed a feed pressure of 30 psi (upper limit for the cassette). Increased pressure can be achieved by tightening clamps slightly to reduce flow (Figure 7B). Make sure clamps are attached to tubing downstream of pressure gauges to register the proper pressure caused by clamping of the tubing. Keep system in this configuration until the reservoir drops to 200 mL, then re-position retentate and filtrate lines back into the reservoir (Figure 7B). Allow the 0.3 M NaOH solution to circulate through both the retentate and filtrate lines for at least 20 min. Notably, for maximum cleaning, aggressive recirculation and increased pressure will help dislodge any contaminants bound to the internal surface of the cassette or tubing. Drain the system by returning the tubing to the setup shown in Figure 7A and proceed to flush the system. Re-fill the tank with 200 mL of sterile Milli-Q water and swish around to rinse NaOH off the sides and top of the reservoir. Clamps should be completely open. Run until ∼5 mL remains in the reservoir and stop pump. Fill the reservoir with 200 mL of sterile Milli-Q water, swish, and let drain to waste. Repeat this step two more times, making sure to rinse the walls and top of the reservoir. It is important to flush the system thoroughly with water to remove all residual 0.3 M NaOH. It is optional to measure the pH of the waste and continue flushing until the pH matches that of the influent. Finally, prime the lines with PBS to avoid risk for osmotic lysis of NDV. Add 75 mL of PBS and turn pump on. Stop the pump when the reservoir is almost empty.

#### 3.2.3 Purification and Concentration of Virus

Once the TFF system has been cleansed, flushed, and contains sterile PBS in the lines, return the tubing to the setup shown in Figure 7B, which will run liquid through a closed system and allow for detection of leaks. While all liquid is being re-circulated, add depth-filtered virus (∼500 mL) to the reservoir and let run for 5 min. When confident that the system is set up properly and there are no leaks, move the filtrate line to a clean waste bottle and keep retentate line returning to feed tank as shown in Figure 7C. Increasing the flow rate or slightly restricting the flow at the retentate valve will preferentially allow the flow to end up in the waste and allow concentration of the virus to occur more rapidly. However, do not exceed a pressure of 10 psi because this may shear the virus. Run the system until 5–10 mL of allantoic fluid remains in the reservoir, then stop the pump and proceed to the buffer exchange step.

#### 3.2.4 Buffer Exchange and Collecting Virus-Containing Eluent

Buffering virus in a 5% sucrose-PBS solution reduces virus aggregation and helps release virus particles that might be bound to the membrane. Open all the clamps and add 50 mL of 5% sucrose-PBS to the reservoir. Continue to run until there is 5–10 mL left in the reservoir. Stop the pump. Close filtrate lines with a clamp so that only the retentate line is open. Put retentate line into a 15 mL conical tube to collect virus, slow the flow rate, and turn on pump. Slowly drain the line. This will be elution 1. To collect residual virus remaining in the lines, connect retentate back to the reservoir (Figure 7C), add 5 mL of 5% sucrose-PBS to the reservoir, and turn on the pump. Allow liquid to circulate for 5 min and then collect into a new 15 mL tube (elution 2). Notably, elution volumes will vary depending on the length of tubing, which should be kept to a minimum. Perform TCID_50_ to determine the amount of virus in each elution. As an example, in one experiment, elutions 1, 2, and 3 contained a total of 2.44 × 10^10^, 1.94 × 10^9^, and 3.45 × 10^8^ PFUs, respectively. Note that if executed properly, 80%–90% of virus can be recovered in the TFF step.

#### 3.2.5 Cleaning the TFF System

Clean the TFF system with 0.3 M NaOH by repeating the flushing procedures outlined in the Sanitizing the Cassette and Tubing section and shown in Figure 7A. The system should be cleansed for at least 1 hr, but can be left to circulate overnight as a closed system (Figure 7B) if more robust cleansing is needed.

#### 3.2.6 Storing the Membrane

Centramate cassettes are reusable. Place the cassette and gaskets into a ziplock bag containing a sufficient amount of 0.3M NaOH storage buffer to cover the cassette completely and store at 4°C. Storage buffer should be changed once a month to prevent the growth of mold. Store tubing in 1 M NaOH.

### 3.3 Sucrose Gradient Purification

**Figure 8:**
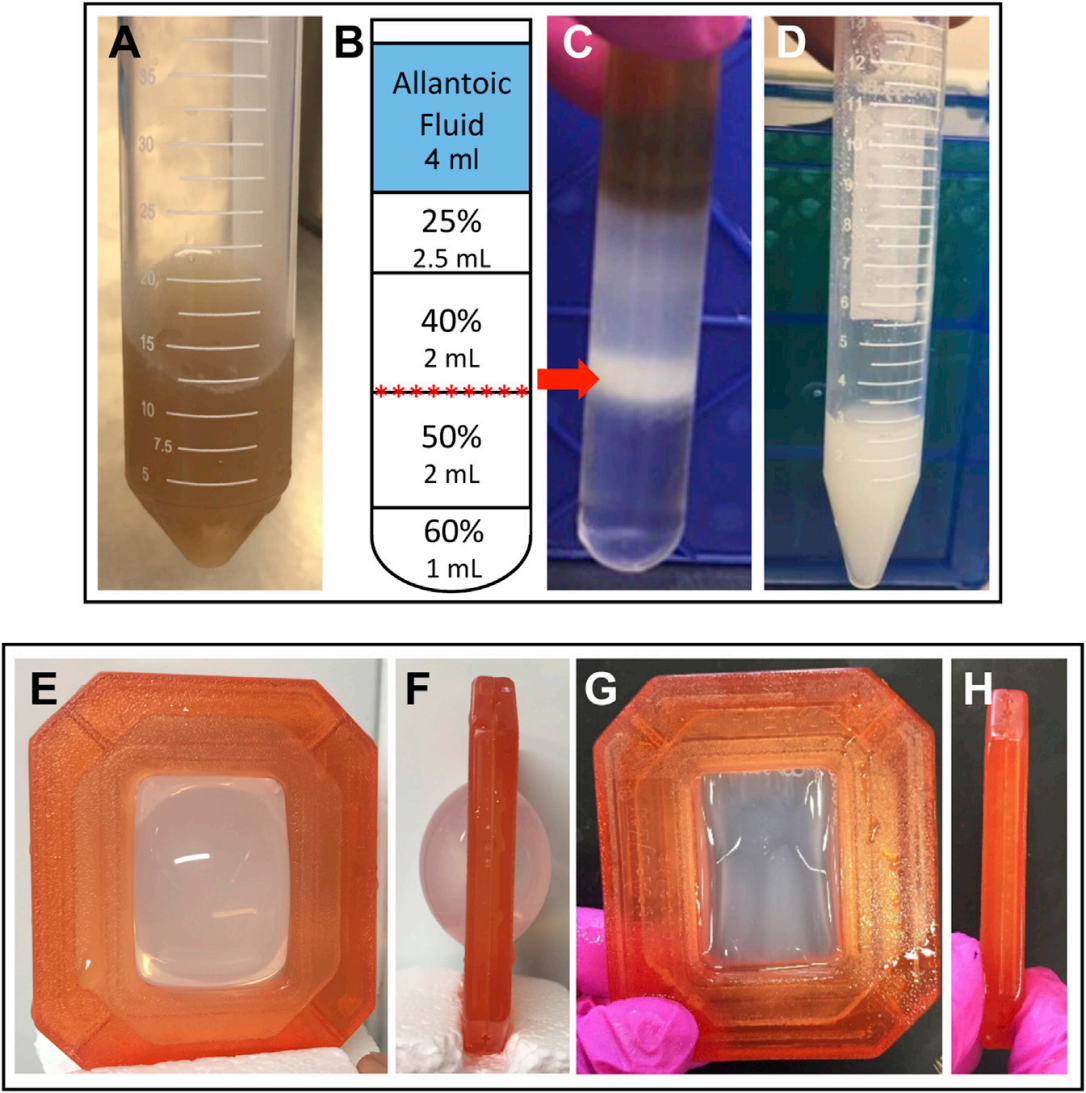
Gradient Ultracentrifugation and PEG Precipitation of NDV after TFF (A) Concentrated allantoic fluid after tangential flow filtration. Fluid appears murky brown because of the concentrated nature of the sample. (B) Schematic of sucrose gradient setup. Red stars indicate where NDV will band after ultracentrifugation. (C) Example of sucrose gradient after ultracentrifugation. NDV is typically located in the opaque white band between 40% and 50% sucrose. (D) Pooled virus recovered from the sucrose gradient. (E) Front and (F) side view of 10K MWCO Slide-A-Lyzer cassette filled with purified NDV, which has increased 3-fold in size after three buffer exchanges in PBS. (G) Front and (H) side view of dialysis cassette after being incubated in 40% polyethylene glycol 20,000 (PEG-20) for 2.5 hr.

Prepare 60%, 50%, 40%, and 25% w/v sucrose in Milli-Q water, 0.22 μM filter-sterilize, and store at 4°C. Prepare sucrose gradients in four 13.2 mL ultracentrifuge tubes. Add 1 mL of 60% w/v sucrose to the bottom of the tube. Carefully layer 2 mL, 2 mL, and 2.5 mL of 50%, 40%, and 25% w/v sucrose, respectively. Layer 4 mL of concentrated NDV (after TFF; Figure 8A) on top of the gradient (Figure 8B). Ultracentrifuge gradients at 120,000 × *g* in a SW41 Ti rotor for 3.5 hr at 4°C. The virus typically bands between the 40% and 50% sucrose layers (Figure 8C). To collect virus, clamp tube to a retort stand and wipe the outside of the tube with 70% ethanol. Place a wide-mouth container below the ultracentrifuge tube to collect the liquid waste that pours out after removal of the syringe. Position an 18G needle attached to a 3 cc syringe to the side of the tube around 5 mm below the virus-containing band. Using a slow, twisting motion, apply pressure as you twist the syringe back and forth until you pierce the tube. Angle the needle, bevel up, toward the interface between the 50% sucrose layer and the virus band and slowly withdraw the virus (∼2 mL). Remove the syringe and allow the rest of the liquid to drain into the container below.

#### 3.3.1 Dialysis and Concentration with Polyethylene Glycol

Because concentrated sucrose can be toxic if delivered intravenously, excess sucrose should be removed from the virus sample. Using a 21G blunt needle, inject up to 8 mL of pooled virus recovered from the sucrose gradient (Figure 8E) into a pre-soaked (2 min in PBS) 10K MWCO dialysis cassette and dialyze in 2 L of PBS at 4°C while stirring. After 2 hr, replace buffer with 2 L of fresh PBS and dialyze for 16 hr as before. Replace buffer with 2 L of fresh PBS and dialyze for an additional 4 hr. Due to the hygroscopic nature of sucrose, the solution in the dialysis cassette frequently doubles or triples in volume (Figures 8E and 8F). To reduce the sample volume, place the dialysis cassette into a small ziplock bag containing 25 mL of pre-chilled 40% (w/v) PEG 20,000. Incubate at 4°C until a volume of ∼2–5 mL is achieved (usually within 4 hr; Figures 8G and 8H). Remove the cassette from the bag and rinse carefully with PBS to eliminate PEG from the surface and from all of the ports. Insert a clean syringe attached to a 21G blunt needle into a different port from that used to load the virus and carefully discharge ∼5 mL of air into the cassette to separate the membranes prior to withdrawing the virus. Carefully remove virus from the dialysis cassette and measure the volume. To retrieve the maximum amount of NDV and to prevent aggregation of virus particles, rinse the cassette twice with 250 μL of a solution of sucrose-PBS that will give a final concentration of 5% sucrose, which if administered neat to mice is not toxic.^25^ For example, if 4.5 mL of virus is recovered from the dialysis cassette, rinse the cassette twice with 250 μL of 50% sucrose-PBS. Dispense purified virus into “single-use” aliquots and store at −80°C.

### 3.4 Confirmation of In Vivo-Grade Purity

The institutional Animal Care Committee at the University of Guelph in accordance with Canadian Council on Animal Care (CCAC) guidelines approved experiments involving mice. To confirm that the virus is pure enough to be safely delivered intravenously, inject at least half a log more than the desired experimental dose into the tail vein of four 8-week-old BALB/c mice, which tend to be more sensitive to virus-induced toxicities than other strains of mice, and monitor for adverse events, including excessive weight loss (>20%), ruffled coat, hunched appearance, apathy, and respiratory distress for up to 4 days post-administration.

### Timing

#### Rescuing Virus (11–12 Days)

• Day 0: Place eggs in incubator.
• Day 4: Seed cells.
• Day 5: Infect/transfect and change media at day’s end.
• Day 6: Co-culture.
• Day 9: Candle eggs, harvest rescued virus (cells+supernatant), and inject eggs.
• Days 10/11: Candle eggs for signs of death.
• Day 11: Move remaining eggs to 4°C at 96 hr.
• Day 11 or 12: Harvest allantoic fluid and perform HA assay.

#### Limiting Dilution and Removal of MVA-T7 (13 Days)

• Day 1: Filter rNDV-containing samples of allantoic fluid and set up dilution series, candle, and inject virus into eggs.
• Days 2/3: Candle eggs for signs of death.
• Day 3: Move eggs to 4°C.
• Day 3 or 4: Harvest allantoic fluid and perform HA assay.
• Day 4: Infect BHK-21 cells.
• Day 7: Collect cells and extract DNA; then perform PCR to confirm absence of MVA-T7.
• Day 8: Set up TCID_50_ to determine titer of MVA-T7-free NDV sample.
• Day 13: Score TCID_50_ plate, aliquot virus, and store at −80°C.

#### Amplifying Virus (4 Days)

• Day 1: Dilute virus to 500 PFUs/100 μL, candle, and inject eight dozen eggs.
• Days 2/3: Check eggs for death.
• Day 3: Move eggs to 4°C between 54 and 60 hr.
• Day 3 or 4: Harvest eggs, HA assay, and clarify allantoic fluid.

#### Purification (3 Days)

• Day 1: Clarify allantoic fluid, depth filter, and TFF purification
• Day 2: Sucrose gradient, first dialysis, second dialysis
• Day 3: Third dialysis, PEG concentration

#### Confirmation of *In Vivo*-Grade Purity (5 Days)

• Day 1: Inject virus into mice.
• Days 1–5: Monitor mice for signs of toxicity.

### Troubleshooting

#### Purification of the rNDV Genome Plasmid Using Columns Can Result in Shearing

Column-purified plasmids should be used for rNDV rescue to maximize transfection efficiency. We found that the GenElute HP plasmid Maxiprep kit (Sigma) was the only kit that did not shear the NDV genome plasmid. For large plasmids, like the NDV genome plasmid, grow bacterial cultures at 30°C in the presence of carbenicillin rather than ampicillin, and culture bacteria for 24 hr rather than the usual 16 hr. If shearing of the genome plasmid continues to occur, purify plasmid DNA by classical mini-prep, including two phenol-chloroform extractions, and confirm that plasmid DNA is intact by gel electrophoresis.

#### Appropriate Number of Infectious Virions to Inject into Eggs for Large-Scale Amplification

Many protocols recommend injecting eggs with 100 PFUs of NDV; however, if the titer is slightly inaccurate, it is possible to inject too little virus. Alternatively, it is important to avoid injecting too much virus because the eggs may die prematurely. We found that injecting 9-day-old embryonated chicken eggs with 500 PFUs NDV/egg in 100 μL ensured that each egg got infected and yielded high-titer virus within the 45–50-hr incubation period.

#### Poor-Quality Allantoic Fluid Can Dramatically Hinder the Purification Process

To obtain the cleanest, highest titer allantoic fluid possible, move eggs to 4°C between 54 and 60 hr post-inoculation, rather than waiting for the embryos to die and membranes to decompose. This will ensure that the allantoic fluid is free of contaminants, such as yolk, which can lead to increased pressure during TFF and/or fouling of the Centramate cassette.

#### Pressure

NDV is vulnerable to shearing at high pressure; therefore, it is important to include pressure gauges during depth filtration and TFF, and to maintain a pressure between 5 and 10 psi. If pressure builds up during depth filtration or TFF, add sucrose to the feed to a final concentration of 5% to reduce aggregation of virus particles.

#### Buildup of Debris on the Centramate Cassette Leads to Increased Pressure

If maintained properly, Centramate cassettes are re-usable; however, over time, buildup on the cassette can increase pressure. Normally 1 hr of cleansing in 0.3 M NaOH is sufficient; however, if you suspect that increased pressure during TFF is due to fouling of the cassette, cleanse the cassette with 0.5 M NaOH for 1 hr followed by an overnight cleanse in 0.3 M NaOH to promote removal of debris from the surface of the cassette.

#### Storing NDV

To avoid multiple freeze-thaws during the virus purification procedure, NDV can be stored at 4°C for up to 3 days. Storing virus in 5% sucrose-PBS prevents virus particle aggregation^15^ and facilitates more accurate determination of virus titers.

### Anticipated Results

Here, we describe a comprehensive protocol for producing high-titer, ultra-clean NDV suitable for intravenous delivery to mice at doses of ≥1 × 10^9^ PFUs, representing a significant improvement over other published methods.^14^, ^26^ When followed correctly, this optimized protocol routinely results in virus titers of 2–5 × 10^9^ PFUs/mL in a volume of 2–6 mL from a starting volume of 500 mL of allantoic fluid. In our hands, this protocol was successful in purifying a strain of NDV that is commonly used as an oncolytic agent; however, it should be possible to purify any strain of NDV using this protocol.

Using this protocol, it is possible to recover approximately ∼60% of input virus. We report percent loss at each step in the purification protocol (Figure 1B) to highlight where improvements can be made and to identify steps that would be suitable for large-scale process development. The superior quality of the virus obtained with this method allows for the administration of up to 1 × 10^9^ PFUs NDV intravenously without any adverse or toxic effects. Indeed, a majority of reports evaluating the oncolytic or immunotherapeutic activity of NDV in preclinical mouse models of cancer administer NDV-containing allantoic fluid diluted in PBS, but it is unclear to what extent the virus has been diluted,^5^, ^27^, ^28^, ^29^, ^30^, ^31^ particularly because the average titer of NDV in allantoic fluid ranges from high 10^7^ to low 10^8^ PFUs/mL.^32^, ^33^, ^34^, ^35^ Not only is NDV harvested from allantoic fluid contaminated with host cell proteins, but the relatively low titers limit the amount of virus that can be delivered at one time. For these reasons, multiple low-dose intratumoral injections (avoiding the bloodstream) over many days are required.

Although the purity and titer of NDV obtained using this method is sufficient for preclinical studies, there is still residual DNA (<0.5 ng/μL) present in the final purified product that would need to be removed before scaling up production for use in clinical trials.

## Author Contributions

L.A.S., S.K.W., and B.W.B. conceived the experiments. L.A.S. and T.M.M. conducted the experiments. L.A.S. and S.K.W. wrote the manuscript with contributions from all other authors (G.A.W., P.P.M., J.J.P., L.S., B.W.B., and T.M.M.).

## Conflicts of Interest

The authors declare no competing financial interests.
